# A Rare Cause of Pancytopenia in Systemic Lupus Erythematosus (SLE) in a Young Patient

**DOI:** 10.7759/cureus.63032

**Published:** 2024-06-24

**Authors:** Muhammad Atif Ameer, Muhammad Ali Tariq, Sarmad Zain, Ahmad Kabir, Muznay Khawaja

**Affiliations:** 1 Internal Medicine, Prime Health Care, Norristown, USA; 2 Rheumatology, Montefiore Medical Center, Jack D. Weiler Hospital (Einstein Campus), New York, USA

**Keywords:** sledai score, sle and hematological association, acute promyelocytic leukemia (apml), sle pathogenesis, sle

## Abstract

Systemic lupus erythematosus (SLE) is an autoimmune condition characterized by antibodies targeting nuclear and cytoplasmic antigens. It can present with diverse clinical symptoms, including pancytopenia. We present the case of an African American woman in her 20s, with a history of SLE who presented with bruising on her body. She had been receiving treatment with hydroxychloroquine, mycophenolate, prednisone, and lisinopril. During a follow-up visit, her workup revealed pancytopenia, prompting an investigation for causes. A flare-up of underlying SLE or mycophenolate toxicity was the likely culprit. However, the clinical picture was not aligned with either. A bone marrow biopsy ultimately led to the diagnosis of acute promyelocytic leukemia. The incidence of acute promyelocytic leukemia following SLE is exceedingly rare. Hence, it could present a significant diagnostic dilemma in patients with pancytopenia and underlying SLE.

## Introduction

Hematological abnormalities are frequently encountered among patients with systemic lupus erythematosus (SLE). These hematological anomalies are predominantly anemia, leukopenia, lymphopenia, and thrombocytopenia [1]. While isolated cytopenias are more common, instances of pancytopenia have also been rarely reported in the literature. In our case report, we present a case in which a patient diagnosed with SLE exhibited pancytopenia during the workup for the history of bruising in the patient.

Pancytopenia in patients with SLE may originate from the underlying disease itself, especially during lupus flares, or it may be due to the effect of immunosuppressive or disease-modifying therapy. The underlying mechanism is not entirely understood. Cytopenias in SLE are often due to peripheral immune-related destruction, which is then associated with marrow normocellularity as evidenced by symptoms of serositis, arthritis, and anemia [2]. Effective management of pancytopenia in patients with SLE necessitates investigating the root cause of the pancytopenia. A thorough bone marrow examination can help if the usual causes have been excluded [3]. Acute promyelocytic leukemia (APML) is a very rare cause of pancytopenia in patients with SLE, and our case highlights one such instance where APML was the cause of pancytopenia, as opposed to the common causes of this finding in this patient population.

## Case presentation

A 28-year-old female with a known history of hypertension, stage 1 chronic kidney disease (CKD), and hypothyroidism (on levothyroxine) presented to the rheumatology office for a follow-up. Six years ago, she was diagnosed with SLE, as evidenced by an SLE disease activity index (SLEDAI) of 6 points, which included symptoms like arthritis, anemia, arthralgia, lupus nephritis IV to V, and mild interstitial fibrosis. At the time of diagnosis, her serological tests revealed positive antinuclear antibody (ANA) (>320 speckled patterns), anti-double-stranded DNA (dsDNA), anti-Smith, anti-Ro (SSA), anti-La (SSB), ribosomal p antibody, as well as hypocomplementemia and proteinuria. In response, she was initiated on a treatment regimen consisting of mycophenolate, hydroxychloroquine, lisinopril, and prednisone. However, she later discontinued the prednisone to reduce her pill burden.

Recently, the patient presented to the rheumatology office for follow-up with bruising on her body, and subsequent blood work showed pancytopenia. Despite discontinuing mycophenolate for a month, her pancytopenia condition did not improve. At this time, considering a likely lupus flare, prednisone 20 mg daily was restarted, but it also showed no improvement in her pancytopenia. This alarming finding of low hemoglobin of 5.6 g/dL led to her admission to the emergency department for a blood transfusion. Initially, the drop in cell counts was suspected to be an SLE flare, but her anti-dsDNA, anti-Smith, anti-U1RNP, anti-Ro/SSA, and anti-La/Ro at this time returned normal, and complement levels were within the normal range. Laboratory values included a sedimentation rate of 38 mm/hour, C-reactive protein at 1.4 mg/L, positive dsDNA antibody assay at 96 IU/mL (normal), and negative antiphospholipid antibodies. C3 and C4 complements were measured normal at 106 and 32 mg/dL, respectively. Lactate dehydrogenase (LDH) was 159 U/L, uric acid was 5.3 mg/dL, haptoglobin was 209 mg/dL, B12 was 1,500 pg/mL, and folate was 5.8 ng/mL. The direct Coombs test was negative, and tests for viral hepatitis (B and C viruses) and HIV serology were negative as well.

During history-taking, the patient denied any chest pain, fever, chills, shortness of breath, or any other constitutional symptoms. During the physical examination, the patient was conscious, with a Glasgow Coma Scale (GCS) score of 15/15. She exhibited severe pallor, discolored conjunctiva, petechiae on the oral mucosa, and bruises on her chest and venipuncture site. Her temperature was 98.2 °F, blood pressure 109/72 mmHg, respiratory rate 18 cycles per minute, and SpO2 100% on room air. Her pulse rate was 87 beats per minute, and cardiopulmonary auscultation was normal. There was no evidence of skin or mucosal ulcers, lymphadenopathy, or hepatosplenomegaly. Peripheral pulses were present and symmetrical.

Blood tests revealed anemia with a hemoglobin level of 5.9 g/dL, thrombocytopenia with platelets at 9 k/μL, and leukopenia with a white blood cell (WBC) count of 1.7 k/μL, with neutrophils at 10% and lymphocytes at 27%. Given the extent of the anemia, transfusions were ordered. After receiving 2 units of packed RBCs and a unit of platelets, her hemoglobin levels increased to 8.4 and 8.7 g/dL, and her platelet count improved to 12, 43, and ultimately 58 k/μL. However, her WBC count continued to deteriorate to 1.6, 0.5, and ultimately 0.4 μL (Table 1).

**Table 1:** Patient's laboratory values for hemoglobin, platelets, and white blood cells (WBCs) at different stages. Initial values before any transfusion, after the first transfusion (packed RBCs), after the second transfusion (packed RBCs), and after the third transfusion (platelets). The differential count of neutrophils and lymphocytes is also included at each stage. As the WBC count decreased, there was a shift in the differential count, with an increase in neutrophils and a decrease in lymphocytes.

Laboratory values	Normal range	Initial	After transfusion 1: packed red blood cells (RBCs)	After transfusion 2: packed RBCs	After transfusion 3: platelets
Hemoglobin (g/dL)	13.5-17.5	5.9	8.4	8.7	8.6
Platelets (k/μL)	150 -450	9	12	43	58
WBCs (k/μL)	4.50-11.00	1.7	1.6	0.5	0.4
Neutrophils (%)	6.4-60	10	10	27	43
Lymphocytes (%)	1.2-35	27	27	57	51

The blood smear displayed elliptocytes and teardrop cells. This presented a diagnostic challenge since mycophenolate-induced pancytopenia and SLE flare were unlikely. The next step was to exclude other sinister causes of pancytopenia, such as bone marrow fibrosis, myelodysplasia, autoimmune marrow failure, differentiation syndrome, viral or fungal infections, and hematological malignancies. To accomplish this, a comprehensive infection screen was performed, which consisted of quantitative screening for Epstein-Barr virus (EBV), cytomegalovirus (CMV), parvovirus B19, and blood and urine cultures. All results came back negative. Therefore, a bone marrow aspiration biopsy was planned, which subsequently showed Auer rods and immature granulocytes with maturation arrest in the myelocytic stage. Cytogenetics revealed abnormal karyotype with t(15;17) (q24.1;q21.2) anomaly. Promyelocytic leukemia/retinoic acid receptor alpha (PML/RARA) gene translocation DNA probe revealed a fusion of PML and RARA loci in 2 of 100 interphase nuclei, confirming the anomaly in chromosome studies. Thus, a diagnosis of APML was made.

The patient received treatment with all-trans retinoic acid/arsenic trioxide (ATRA/ATO) for APML, and she tolerated the treatment well. Throughout the treatment, she developed differentiation syndrome. Her regular medications, including lisinopril, hydroxychloroquine, levothyroxine, and aspirin, were continued throughout her treatment. Due to the potential for QT interval prolongation with arsenic therapy and hydroxychloroquine, serial electrocardiograms (EKGs) were performed to monitor cardiac function. During her second cycle of consolidation, she developed proteinuria with a urine protein-to-creatinine ratio of 2,500. The urine analysis showed no sediments, complements were within normal range, and anti-dsDNA was normal at 22. Since isolated high-grade proteinuria was not a classic presentation of differentiation syndrome, this clinical picture was deemed a renal lupus flare, and it necessitates the start of mycophenolate. Clinically, there was no arthritis, rash, or serositis at this stage. Mycophenolate 500 mg twice a day was restarted at this stage after being held for three months, with up-titration to 1,500 mg twice a day, resulting in improved proteinuria. Literature supports that there was no contraindication to using mycophenolate concurrently with the use of ATRA/ATO. The plan for SLE treatment is a continuation of the maximum dose of mycophenolate 1,500 mg twice a day, lisinopril 30 mg daily, and prednisone 10 mg daily with frequent outpatient follow-ups and lab monitoring. For APML, the plan is in place to continue consolidation therapy with ATRA/ATO until remission is achieved.

## Discussion

The development of APML in the setting of SLE is extremely rare. While some malignancies have been linked to SLE, including lymphoid malignancies, APML is a lesser-known association. SLE is a complex autoimmune condition characterized by antibodies targeting nuclear and cytoplasmic antigens, leading to inflammation in multiple organ systems. The disease exhibits diverse clinical symptoms and follows a pattern of recurrent relapses and remissions. On the other hand, APML is a subtype of acute myeloid leukemia (AML) characterized by abnormal promyelocytes in the bone marrow. The prevalence of SLE in the United States is reported to be 20 to 150 cases per 100,000 [4,5]. There has been an increase in the detection of mild SLE cases [6]. This is attributed mainly to increasing awareness about the disease and the availability of better diagnostic tools. The incidence rates are estimated to be 1 to 25 per 100,000 in North America, South America, Europe, and Asia [4].

The development of APML in SLE is a rare occurrence, and the exact incidence of this coincidence remains unknown due to the rarity of SLE itself. However, a few case reports have documented this association in the medical literature. The incidence of APML in patients with SLE is a topic of interest in rheumatology and hematology. The Surveillance, Epidemiology, and End Results (SEER) study shows an increased incidence of myeloid malignancies in SLE, but an exact association of APML in SLE has not been reported. APML in a young patient with SLE represents a diagnostic challenge due to overlapping symptoms of both conditions and ruling out other differentials.

Previous studies have shed light on the potential relationship between SLE and hematologic malignancies. Taguchi et al. reported a case of APML developing in the course of SLE, highlighting the diagnostic challenge and the need for vigilance in monitoring patients with SLE for the development of hematologic malignancies [7]. Additionally, Tahri et al. discussed a rare association between AML and SLE, emphasizing the need for further exploration of this topic [8].

The case underlines the importance of a comprehensive diagnostic approach for persistent pancytopenia in SLE cases. The initial workup involved ruling out a lupus flare and discontinuing potentially contributing medications. The negative lupus panel results and persistent pancytopenia prompted further investigation, leading to the identification of Auer rods in the bone marrow biopsy, PML/RARA translocation, and the subsequent diagnosis of APML.

SLE is known for its wide range of symptoms and hematological abnormalities [9], but the development of APML in a patient with SLE is very rare. The patient initially presented with bruising, which could have been attributed to her lupus or its treatment. However, the subsequent discovery of pancytopenia raised concerns beyond the usual lupus-related hematological issues.

Patients with SLE often experience hematological abnormalities such as anemia, leukopenia, and thrombocytopenia [10]. Pancytopenia in SLE can be due to various causes, including infections, aplastic anemia, myelodysplastic syndromes, myelofibrosis, Fanconi anemia, lupus flare, or immunosuppressive therapies. Negative infectious workup and failure of pancytopenia resolution after weeks of mycophenolate cessation should alert physicians to other possible etiologies of pancytopenia. While leukocytosis is more typical in APML, this case showed an interesting deviation from the norm. The patient initially had a low white cell count with a lymphocytic predominance. Subsequently, a neutrophilic shift was observed. This atypical leukocyte count pattern adds a layer of complexity to the diagnostic process, as it may not immediately lead to the suspicion of leukemia. This highlights the importance of keeping an open mind when presented with unexpected clinical manifestations in patients with autoimmune diseases.

Managing this patient's condition presented a challenge due to the potential interactions between her SLE treatment and the newly prescribed ATRA/ATO therapy for APML. Development of differentiation syndrome with the initial induction phase of ATRA/ATO therapy required close monitoring and treatment with steroids. This depicts the need for physicians to be vigilant. Hydroxychloroquine was permanently discontinued as there is a known risk of QT prolongation with concurrent ATRA/ATO and hydroxychloroquine. A concurrent regimen of mycophenolate and prednisone, along with ATRA/ATO consolidation therapy, did not yield any side effects. Managing both conditions concurrently poses significant challenges. Development of high-grade proteinuria in this case while off immunomodulators for SLE required a multi-disciplinary discussion to determine the exact etiology. The final consensus for the etiology of proteinuria was the renal lupus flare secondary to cessation of Mycophenolate. This highlights the need for collaboration between treating physicians in oncology and rheumatology. 

## Conclusions

Pancytopenia in a patient with SLE is a common finding; however, determining the precise etiology of pancytopenia is important to dictating the right treatment. The causes of pancytopenia in patients with SLE could be medication toxicity, a flare-up of underlying lupus, or the development of a hematologic malignancy. This case highlights the importance of vigilance in monitoring patients with SLE for developing hematologic malignancies.
